# 25-Hydroxyvitamin D and Total Cancer Incidence and Mortality: A Meta-Analysis of Prospective Cohort Studies

**DOI:** 10.3390/nu11102295

**Published:** 2019-09-26

**Authors:** Jianmin Han, Xiaofei Guo, Xiao Yu, Shuang Liu, Xinyue Cui, Bo Zhang, Hui Liang

**Affiliations:** Institute of Nutrition and Health, Qingdao University, Qingdao 266071, China; 18004015886@163.com (J.H.);

**Keywords:** vitamin D, cancer incidence, cancer mortality, prospective study, meta-analysis

## Abstract

Epidemiological studies have suggested inconclusive associations between 25-hydroxyvitamin D and total cancer incidence and mortality. The aim of this study was to quantitatively assess these associations by combining results from prospective cohort studies. A systematic literature search was implemented in PubMed and Scopus databases in April 2019. Comparing the highest with the lowest categories, the multivariate-adjusted relative risks (RRs) and the corresponding 95% confidence intervals (CIs) were pooled using a random-effects model. A trend estimation was performed using a two-stage, dose-response, meta-analysis method. Twenty-three independent prospective studies were included for data synthesis. Eight studies investigated the association between 25-hydroxyvitamin D and the risk of cancer incidence (7511 events and 70,018 participants), and the summary estimate showed that 25-hydroxyvitamin D is marginally associated with cancer risk (Summary RR = 0.86; 95% CI: 0.73, 1.02; I^2^ = 70.8%; *p* = 0.001). Sixteen studies investigated the association between 25-hydroxyvitamin D and the risk of cancer mortality (8729 events and 101,794 participants), and a higher 25-hydroxyvitamin D concentration was inversely associated with the risk of cancer mortality (Summary RR = 0.81; 95% CI: 0.71, 0.93; I^2^ = 48.8%, *p* = 0.012). Dose-response analysis indicated that the risk of cancer incidence was reduced by 7% (RRs = 0.93; 95% CI: 0.91, 0.96), and the risk of cancer mortality was reduced by 2% (RRs = 0.98; 95% CI: 0.97, 0.99), with each 20 nmol/L increment of 25-hydroxyvitamin D concentration. This meta-analysis provides evidence that a higher 25-hydroxyvitamin D concentration is associated with a lower cancer incidence and cancer mortality.

## 1. Introduction

Cancer is the leading cause of global morbidity and mortality, with approximately 18.1 million new cancer cases and 9.6 million cancer deaths in 2018 [1]. The number of people who died of cancer ranks second, second only to the number of deaths from cardiovascular and cerebrovascular diseases worldwide [2,3]. In recent years, great progress has been made in the prevention and treatment of certain cancers [4,5,6]. Despite these advances, the burden of cancer is increasing due to the increasing global population and ageing, as well as risk factors, such as smoking, obesity and unhealthy dietary patterns. Therefore, it is necessary to identify many aspects of the beneficial factors for cancer prevention.

As a secosteroid hormone, the biologically active metabolite of vitamin D is attributed to 1,25-dihydroxyvitamin D (1,25(OH)2D), which is synthesized from the circulating 25-hydroxyvitamin D (25(OH)D) by 1a-hydroxylase (CYP27B1). The classical biological function of vitamin D is to regulate calcium and phosphorus balance and maintain bone health. Recent studies have found that it also plays an important role in anti-inflammatory, anti-fibrosis and immunoregulation processes, and it is also involved in maintaining the growth and development of the body and regulating cell proliferation, differentiation and apoptosis [7,8,9]. As early as 1980, an epidemiological study proposed that vitamin D may have anti-cancer properties [10]. Since then, numerous studies have supported this hypothesis [11,12,13]. The anti-cancer effect of vitamin D might be related to its regulation of cell proliferation and differentiation, growth factor gene expression, and signal transduction and apoptosis [14,15,16,17,18,19]. Extensive investigations, both in vitro and in vivo, have demonstrated that vitamin D is associated with cancer [20,21,22,23]. In addition, there is strong evidence, from cell experiments and animal studies, that supports the anti-tumor effects of vitamin D [14,24,25]. However, the results from prospective cohort studies have been inconsistent.

In fact, there were already several meta-analyses of prospective studies about cancer incidence or mortality, existing meta-analysis also yield inconsistent results [26,27,28,29]. Several prospective cohort studies recently explored the associations between 25-hydroxyvitamin D and cancer risk and mortality, while the results remain controversial [30,31,32,33]. However, the meta-analysis of prospective studies has not been updated in recent years. Therefore, to provide a more comprehensive, up-to-date assessment of the associations between 25-hydroxyvitamin D and cancer risk and mortality, we conducted a systematic review and meta-analysis to quantitatively assess these associations. In addition, a dose-response meta-analyses were performed to explore the trend estimation.

## 2. Materials and Methods 

### 2.1. Search Strategy and Inclusion Criteria

A systematic literature search was performed in the Cochrane library, Embase and PubMed databases until April, 2019 using the search query: Vitamin D, VD, 25-hydroxyvitamin D, 25(OH)D, 1,25-dihydroxyvitamin D or 1,25(OH)2D, paired with cancer, tumor, neoplasm, mortality, incidence, risk, and occurrence.

Two authors independently performed the literature search and identified potential studies from the title, abstract and full-text. The inclusion criteria were prospective studies (comprising the prospective cohort and, nested case-control studies). The exposure of interest was the serum 25(OH)D concentration or plasma 25(OH)D concentration, and the end point of interestwas the number of cancer patients or deaths due to cancer during follow-ups.

### 2.2. Data Extraction and Quality Assessment

For the identified studies, data extraction was conducted by two investigators, and any discrepancy was resolved via discussion. The characteristics, including the first author’s surname, publication year, geographic region, duration of follow-up, mean age at the baseline, number of participants and cases, age of the participants, relative risk (RR), with the corresponding 95% confidence interval (CI), and the assay used to measure 25(OH)D, and covariates, adjusted for in the analyses, were extracted from the eligible studies. While most of the original studies used the lowest dose group as the reference group, a few used other groups as the reference group, so we converted this kind of study into the lowest dose group, as the reference group [34]. The Newcastle-Ottawa Scale criteria, with a 9-star system, was adopted to perform the quality assessment. The full score was defined as 9 stars, and a study was classified as low, moderate and high-quality using 0–3, 4–6 and 7–9 stars, respectively [35]. The assessments were conducted by two investigators, any discrepancy was resolved with the third investigator (Hui Liang) to reach agreement.

### 2.3. Statistical Analysis 

RR was regarded as the common risk estimate for the association between 25(OH)D and cancer risk and mortality. Considering that the eligible studies were prospective studies, thus, odds ratio (OR) or HR was directly considered as RR for data synthesis. Multivariate-adjusted RRs, with the corresponding 95% CIs for the highest versus lowest category, were logarithm transformed, and the summary RR was calculated using a random-effects model, which was weighted by the inverse of the variance of the RRs. Studies including three or more 25(OH)D categories were eligible for dose-response analysis [36]. The median concentration of 25(OH)D, assigned in respective quantiles, was extracted. The midpoint of the lower and upper categories was regarded as the 25(OH)D concentration of the quantile, if the media concentration was not provided. The concentration of 25(OH)D was defined as 1.2 times higher than the highest boundary, if the highest quantile was open-ended. Meanwhile, the 25(OH)D concentration of the lowest quantile (the reference) was set as 0 in each study [37]. We performed a random-effects, dose-response, meta-analysis to estimate the potential curvilinear relation. A two-stage, random-effects, dose-response analysis was carried out to estimate the associations between 25-hydroxyvitamin D and cancer incidence and mortality. To estimate the potential curvilinear (non-linear) associations between 25-hydroxyvitamin D concentration and cancer risk and cancer mortality, a restricted cubic spline model was applied, with 3 knots at fixed percentiles of the 25-hydroxyvitamin D subclasses distribution (25%, 50% and 75%) [38]. P for non-linearity is obtained by testing the null hypothesis that the regression coefficients of the spline transformations are all equal to zero [39]. I^2^ statistic was used to estimate the heterogeneity between studies. To examine the potential sources of heterogeneity, subgroup and univariate meta-regression analyses, including duration of follow-up, region, study quality and mean age of participants, was implemented based on the information of these studies. In order to assess whether a study had an undue influence on the summary estimates, we deleted one study at a time and recalculated the summary estimates. The Egger’s test was used to assess publication bias (*p* < 0.1 [40] is significant). Statistical analysis was performed using STATA 11.0 (STATA CORP, College station, TX, USA). The *p* value was two-tailed and the significance level was 0.05.

## 3. Results 

### 3.1. Literature Search and Study Characteristics

**Figure 1:**
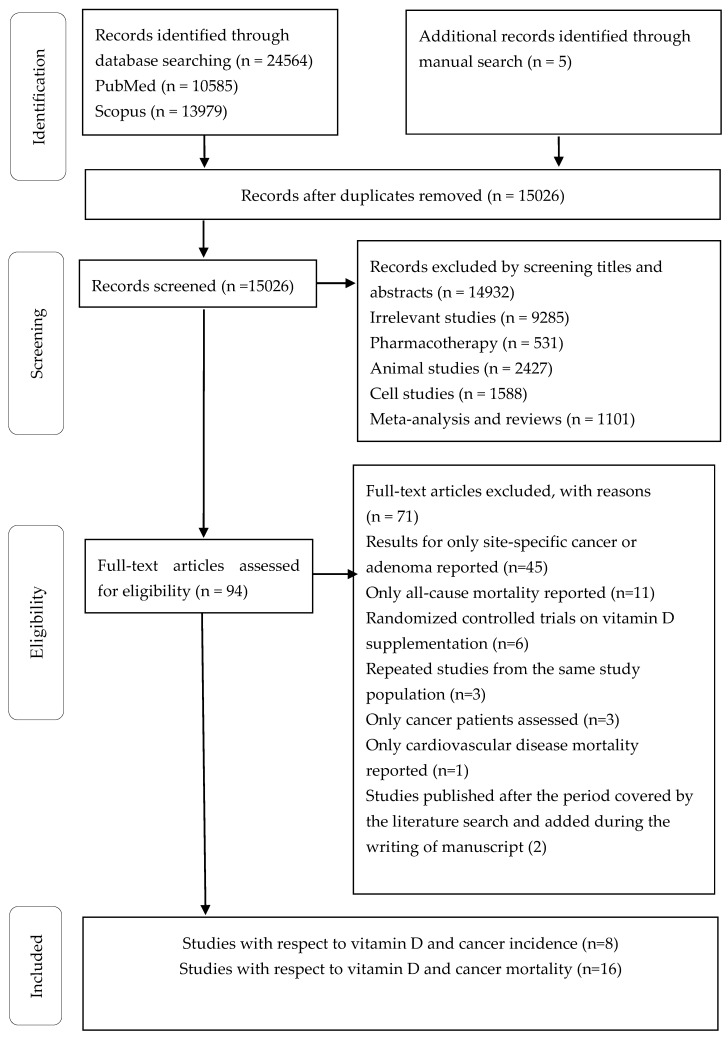
Flow chart of the study selection process.

The flow chart of the literature search is presented in Figure 1. We identified 10,585 citations from PubMed and 13,979 from Scopus. Among these, 15,026 citations remained, after the exclusion of duplicates, and 14,932 citations were excluded by screening the titles and abstracts, leaving 94 articles for full-text examination. Of these, 71 articles were excluded, because they did not satisfy the inclusion criteria (e.g., they did not provide sufficient data, an adequate study design or a relevant review). Finally, 23 articles were included for data synthesis.

Basic information concerning the eligible studies is listed in Table 1 and Table 2. A total of 8 prospective cohort studies were included in the meta-analysis of total cancer incidence (7511 events and 70,018 participants) [13,30,31,32,41,42,43,44], and 16 prospective cohort studies were included in the meta-analysis of total cancer mortality (8729 events and 10,794 participants) [33,43,45,46,47,48,49,50,51,52,53,54,55,56,57,58]. Eight prospective cohort studies were conducted in the US [44,47,48,50,52,55,57], thirteen were performed in Europe [13,32,33,41,42,43,45,49,51,53,54,56,58], and the remaining studies were executed in Asia [30,31,46]. The level of covariate adjustment in the individual studies differed, but all studies adjusted for age, sex and some indicators relating to tumor spreading. Most studies measured the 25(OH)D serum concentration with immunoassays [30,32,41,46,48,50,53,55,57] or mass spectrometry [31,42,43,44,47,49,52,56]. Besides, other studies measured 25(OH)D serum concentration using a radioimmunoassay [13,45,51] and competitive protein-binding assay [33,54,58]. Five studies were limited to males [13,33,43,45,48] and two to females [47,52]. On the basis of the Newcastle-Ottawa scale criteria, 15 prospective studies were classified as high-quality [13,30,31,41,46,47,49,50,51,53,54,55,56,57,58], and the remaining studies were classified as moderate-quality [32,42,43,44,45,48,52] (Supplementary Tables S1 and S2). 

**Figure 2:**
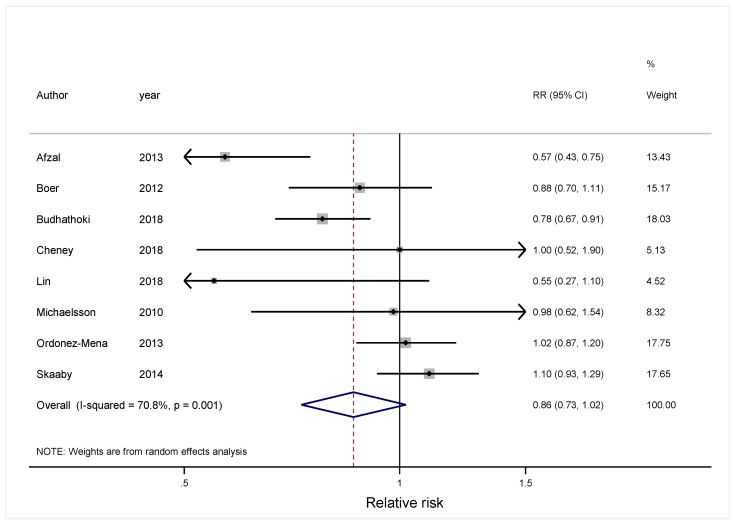
Forest plot to quantify the association between 25-hydroxyvitamin D and total cancer incidence. The summary relative risk was calculated using a random-effects model. The diamonds denote the summary risk estimate, and the horizontal lines represent 95% confidence interval (CI). Abbreviations: RR, summary relative risk.

**Figure 3:**
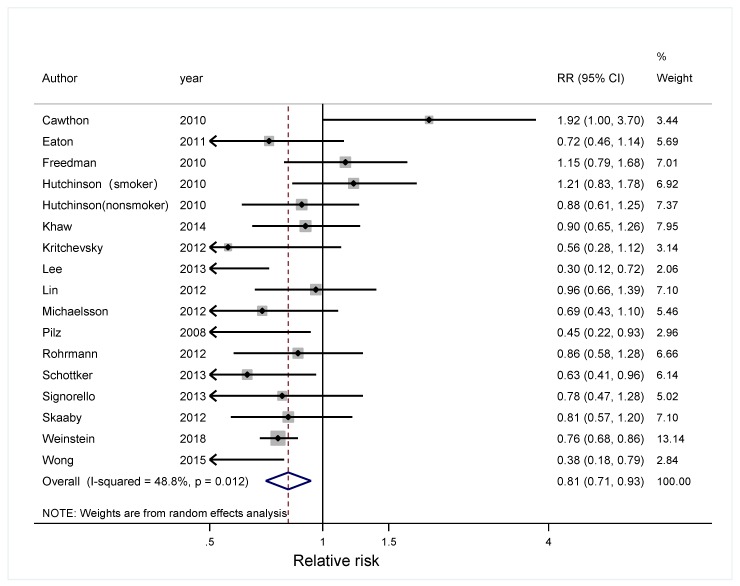
Forest plot to quantify the association between 25-hydroxyvitamin D and total cancer mortality. The diamonds denote the summary risk estimate, and the horizontal lines represent 95% CI. Abbreviations: RR, relative risk.

**Figure 4:**
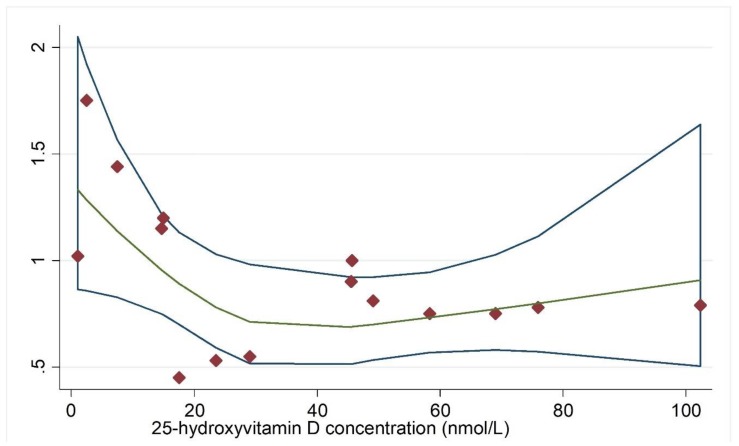
Dose-response analysis for vitamin D concentration and cancer incidence. Adjusted RRs from each exposure quantile of 25(OH)D concentration in included individual studies were represented by the diamonds, and corresponding intervals (CIs) were represented by the blue trendline. Abbreviations: RR, relative risk. The green line indicated the dose-response linear trend between 25(OH)D concentration and risk of cancer incidence by use of variance-weighted least squares regression of fixed effect model.

**Figure 5:**
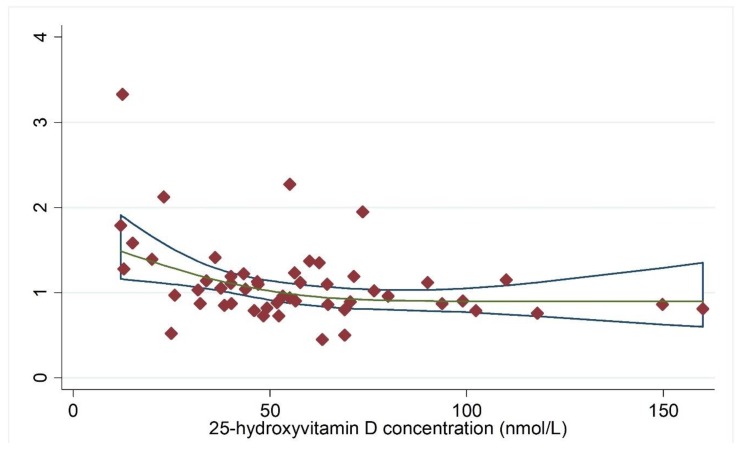
Dose-response analysis for vitamin D concentration and cancer mortality. Adjusted RRs from each exposure quantile of 25(OH)D concentration in included individual studies were represented by the diamonds, and corresponding intervals (CIs) were represented by the blue trendline. Abbreviations: RR, relative risk. The green line indicated the dose-response linear trend between 25(OH)D concentration and risk of cancer mortality by use of variance-weighted least squares regression of fixed effect model.

In the meta-analysis combining results of eight independent cohort studies [13,30,31,32,41,42,43,44], 25(OH)D was marginally associated with cancer risk, with a significant between-study heterogeneity (Summary RR = 0.86; 95% CI: 0.73, 1.02; I^2^ = 70.8%; *p* = 0.001) (Figure 2). Regarding total cancer mortality, the summary estimate found an inverse association, with a significant between-study heterogeneity [33,43,45,46,47,48,49,50,51,52,53,54,55,56,57,58] (Summary RR = 0.81; 95% CI: 0.71, 0.93; I^2^ = 48.8%, *p* = 0.012) (Figure 3). Dose-response analysis indicated that the risk of cancer incidence was reduced by 7% (RRs = 0.93; 95% CI: 0.91, 0.96) (Figure 4), and the risk of cancer mortality was reduced by 2% (RRs = 0.98; 95% CI: 0.97, 0.99) for each 20 nmol/L increment of 25-hydroxyvitamin D concentration (Figure 5). 

### 3.2. Subgroup Analysis

In terms of total cancer incidence, subgroup analysis, stratified by region, indicated that the pooled effect of 25-hydroxyvitamin D was significantly associated with cancer incidence in Asia [30,31] (Pooled RR = 0.77; 95% CI: 0.66, 0.89; I^2^ = 76.0%; *p* = 0.341), but there was no significant correlation in Europe [13,32,41,42,43] (Pooled RR = 0.91; 95% CI: 0.71, 1.16; I^2^ =0.002; *p* = 0.002). Studies were divided according to gender, and the male group [13,31,42,43] showed a borderline association between 25-hydroxyvitamin D and cancer risk (Pooled RR = 0.75; 95% CI: 0.55, 1.01; I^2^ = 65.0%; *p* = 0.025), but there was no significant correlation in the female subgroup [31,42] (Pooled RR = 0.80; 95% CI: 0.35, 1.82; I^2^ = 75.8%; *p* = 0.042). The studies were stratified by the study quality, indicating that there was a borderline association between 25-hydroxyvitamin D and cancer risk in the high-quality group [13,30,31,41] (Pooled RR = 0.80, 95% CI: 0.63, 1.01), but there was no significant correlation in the moderate-quality group (Table S3). 

Similarly, the summary estimate of 25-hydroxyvitamin D was inversely associated with cancer mortality in the female group [31,47,50,52,56] (Pooled RR = 0.72; 95% CI: 0.52, 0.98; I^2^ = 56.6%; *p* = 0.075), but not in the male group [31,33,43,45,48,50] (Pooled RR = 0.90; 95% CI: 0.73, 1.12; I^2^ = 78.7%; *p* < 0.001). When the articles were divided into subgroups by the study quality, 25-hydroxyvitamin D was negatively correlated with cancer mortality in the high-quality subgroup [33,46,47,49,50,51,53,54,55,56,57,58] (Pooled RR = 0.82; 95% CI: 0.71, 0.95; I^2^ = 48.8%; *p* = 0.058), but there was no significant correlation in the moderate-quality subgroup [34,44,46,53] (Pooled RR = 0.78; 95% CI: 0.52, 1.18; I^2^ = 40.9%; *p* = 0.062). The studies stratified by region showed that there was a significantly association between vitamin D concentration and cancer mortality in Europe [33,43,45,49,51,53,54,56,57] (Pooled RR = 0.79, 95% CI: 0.68,0.90; I^2^ = 38.1%; *p* = 0.104), and a marginal association in the US [48,49,51,53,56,58] (Pooled RR = 0.83; 95% CI: 0.56, 1.21; I^2^ = 48.8%; *p* = 0.012) (Supplementary Table S4).

### 3.3. Sensitivity Analysis and Publication Bias

In a sensitivity analysis, each study was sequentially deleted, and the remaining data were re-calculated. The results showed that the summary estimate was not substantially driven, to the exclusion of any one study. In publication bias analysis, non-significant publication bias was observed using Egger’s regression test (Supplementary Figures S1 and S2).

## 4. Discussion

Analyses of 8 studies on cancer incidence and 16 studies on cancer mortality, showing that 25-hydroxyvitamin D is marginally associated with cancer risk and inversely associated with mortality, were pooled. The dose-response analysis showed that cancer risk decreased by 7% (95% CI: 0.90, 0.95), with a 20 nmol/L increment of 25-hydroxyvitamin D, and cancer mortality decreased by 2% (95% CI: 0.97, 0.99), with a 20 nmol/L increment of 25-hydroxyvitamin D. In fact, a meta-analysis of a prospective cohort study on the relationship between vitamin D concentration and cancer incidence and mortality was performed in 2013 [26]. Based on the summary estimates, the present study confirms the findings presented in the previous meta-analysis. 

This study excluded the study of Giovannucci et al. [59] because it predicted, rather than measured, 25(OH)D concentrations. We also excluded the retrospective cohort study of Krause et al. [60]. Of course, we have supplemented and updated the previous meta-analysis and included more studies [30,31,33,41,45,47,56,57,58]. In addition, a meta-analysis of randomized controlled trials was recently conducted. Goulao et al. [61] concluded that vitamin D supplementation alone reduces the incidence of cancer or cancer mortality, and Keum et al. [62] indicated that vitamin D supplementation significantly reduced total cancer mortality but did not reduce total cancer incidence. The development of cancer is a long-term and gradual process, while the intervention time of randomized controlled trials is limited.

As can be observed in our study, several studies have reported null associations between vitamin D concentration and cancer incidence or mortality [13,30,33,45,47,51,54]. However, other studies have reported modest statistically significant reductions in cancer risk or mortality, with increments of 25-hydroxyvitamin D concentration [31,32,41,42,43,46,48,49,50,52,53,55,56,57,58]. At the same time, studies found that a higher 25-hydroxyvitamin D concentration is marginally associated with the cancer incidence in males, but no correlation was found in females. In terms of cancer mortality, a higher 25-hydroxyvitamin D is inversely associated with the cancer incidence in females, but no correlation was found in males. The reason may be that few studies have focused on the gender segregation. While the inclusive prospective studies have shown controversial and inconsistent associations, the summary estimate provided substantial evidence that a higher 25-hydroxyvitamin D is associated with a lower incidence and mortality. 

The inverse association between 25-hydroxyvitamin D and cancer incidence and mortality is biologically plausible. Existing studies have shown that vitamin D regulates the entire process of tumorigenesis, from initiation to stabilization, and the interaction with the cellular microenvironment [20,23,62]. Vitamin D regulates multiple signaling pathways through its active form, 1,25(OH)2D, and shows a direct effect on cell proliferation, differentiation, and cell death. In addition, vitamin D has been shown to have anti-inflammatory, oxidative stress, and immune response, which may help to inhibit tumor cell initiation and progression [42,43]. These laboratory studies provide possible mechanisms through which vitamin D may be associated with reduced cancer incidence and mortality.

Our meta-analysis may have several limitations which must be taken into account. First, due to the observational nature of the data, it is possible that the observed significant inverse association between 25-hydroxyvitamin D and cancer incidence and mortality could be due to unmeasured or residual confounding. Second, the reduced cancer incidence and mortality may be associated with other health behaviors (e.g., balanced nutrition, adequate sleep, exercise and so on). A further limitation was that the eligible studies varied in several respects, including differences in the study populations, baseline comorbidities, and measurement of the exposure, and it is possible that methodological differences might have confounded the differences recorded across subgroups of the study. Not all prospective studies are conducted in the general population (e.g., the study of Pilz et al. [50] was conducted in the Patients Referred to Coronary Angiography). 

The merits of this research should be emphasized. First, we have included more research than the previous meta-analyses. The large sample size of the meta-analysis provided powerful statistical power for the quantitative evaluation of this association. Second, eligible studies are medium- and high-quality prospective cohort studies that reduce the likelihood of recall errors and selection biases. Third, the confounding factors of the individual studies have been greatly adjusted to minimize biases. Besides, the follow-up of the prospective cohort studies is sufficiently long. Furthermore, dose-response analysis was also conducted to provide substantial evidence that vitamin D concentration is associated with cancer incidence and mortality, with a dose-dependent trend.

## 5. Conclusions

The present study provides compelling evidence that a higher 25-hydroxyvitamin D concentration is marginally associated with cancer incidence and inversely associated with cancer mortality. Since the majority of the prospective studies were performed in Western countries, further prospective studies should be conducted in other regions, with different ethnic origins, to confirm these associations.

## Figures and Tables

**Table 1:** Prospective studies reporting on the association of circulating 25(OH)D serum concentration with total cancer incidence ^a.^

First Author and Cohort	Publication Year and Region	Mean Age (Gender)	Subjects (Cases)	Follow-Up Period	Exposure Measure	Outcome Ascertainment	Covariates Adjusted
Afzal [13], CCHS	2013, Denmark	57.7 (M)	9791 (1081)	28 y	radioimmunoassay	obtained from the Danish Cancer Registry	age, sex, pack-years, BMI, alcohol consumption, leisure time and work-related physical activity, duration of education and month of blood sample
Budhathoki [31], JPHC	2018, Japan	53.7 (Both)	33,736 (3301)	19 y	chemiluminescent enzyme immunoassay	obtained from local hospitals and population-based cancer registries	age, sex, body mass index, smoking, alcohol use, physical activity, family history of cancer, and reported history of diabetes.
Lin [32], CSPPT	2018, China	61.8 (Both)	462 (231)	4.5 y	liquid chromatography tandem mass spectrometry	diagnosed based on either positive pathologic findings or specific clinical manifestations	age, sex, treatment group, and study site, and adjusted for BMI, smoking status, alcohol consumption, baseline SBP and DBP, time-averaged SBP and DBP during treatment, baseline fasting blood glucose, total cholesterol, triglycerides, season of blood collection, plasma calcium levels, folate, vitamin B_12_, vitamin A, vitamin E and vitamin K
Skaaby [42], Monica10, Inter99, and Health2006	2014, Denmark	60.5 (Both)	12,204 (1248)	11.3 y	chemiluminescent enzyme immunoassay	obtained from the Danish Central Personal Register	study group (no intervention (participants from Monica10), lifestyle counseling (group B from Inter99), lifestyle and group counseling (group A from Inter99)), gender, education, season, physical activity, smoking habits, alcohol intake, intake of fish, and BMI
Cheney [33], KORA	2018, Germany	53.5 (Both)	2003 (69)	7 y	enzyme immunoassay	identified using a standardized interview (FF4) by trained medical personnel.	age, sex, BMI, season of blood draw, physical activity, smoking status, smoking status alcohol consumption and vitamin D supplementation
Boer [45], CHS	2012, USA	74.0 (Both)	1621 (335)	15 y	high-performance liquid chromatography tandem mass spectrometry	obtained from available hospital discharge summaries, diagnostic test records, and consultation reports	age, sex, clinical site, smoking, body mass index, and physical activity
Michaelsson [44], ULSAM	2010 Sweden	71 (M)	1194 (373)	12.7 y	high-performance liquid chromatography–tandem mass spectrometry	obtained from Swedish National Cancer Registry and Cause of Death Registry	age, weight, height, calcium intake, season of blood draw, social class, smoking status, leisure physical activity, self-perceived health, diabetes mellitus, other endocrine disease, hematologic diseases, dermatoses, infectious disease, musculoskeletal disease, psychiatric disease, respiratory disease, kidney or urinary disease, gastrointestinal disease, supplemental vitamin D use, total vitamin D intake, fish intake, plasma parathyroid hormone, plasma cystatin C, plasma C-reactive protein, serum calcium, serum phosphate, plasma troponin I, plasma N-terminal pro brain natriuretic peptide, plasma cholesterol, plasma triglycerides, plasma HDL cholesterol, plasma retinol, plasma insulin, total energy intake, and alcohol intake and systolic blood pressure, diastolic blood pressure, lipid-lowering treatment, and antihypertensive treatment.
Ordonez -Mena [27]	2013 Germany	63 (Both)	9007 (873)	8 y	liquid chromatography/tandem mass spectrometry	obtained from Saarland Cancer Registry	Age, sex, multivitamin use, fish consumption, red meat consumption, daily fruit intake, daily vegetables intake, BMI, scholarly education, physical activity, smoking, and family history of cancer

^a^ There were 8 prospective cohort studies comprising 7511 cancer incidence cases among 70,018 participants in relation to 25-hydroxyvitamin D. CCHS: The Copenhagen City Heart Study; JPHC: Japan Public Health Center-based Prospective Study; CSPPT: China Stroke Primary Prevention Trial; SBP, systolic blood pressure; DBP, diastolic blood pressure; BMI: Body mass index; KORA: Cooperative Health Research in the Region of Augsburg study; ULSAM: The Uppsala Longitudinal Study of Adult Men; CHS: Cardiovascular Health Study; HDL: High Density Lipoprotein; y: year; M: male; F: female.

**Table 2:** Prospective studies reporting on the association of circulating 25(OH)D serum concentration with total cancer mortality ^b.^

First Author	Publication Year and Region	Mean Age (Gender)	Subjects (Cases)	Follow-Up Period	Exposure Measure	Outcome Ascertainment	Covariates Adjusted
Lee [46], EMAS	2013, Europe	60 (M)	2816 (28)	4.3 y	radioimmunoassay	Obtained from death certificates, death registers and medical/hospital records.	age, center, smoking status, alcohol consumption, self-reported morbidities, Physical Activity Scale for the Elderly score, Reuben’s Physical Performance Test rating and serum creatinine
Lin [47], GPTL	2012, China	56.5 (Both)	110 (217)	24 y	enzyme immunoassay	Obtained from records of the village doctors, evaluated and verified by a panel of Chinese experts	age and sex, with additional adjustment by separate continuous age variables for each stratum as well as sex, hypertension, tobacco smoking, BMI, and alcohol consumption.
Wong [48], CAIFOS	2015, US	75.1 (F)	1188 (84)	10 y	liquid chromatography tandem mass spectrometry	Obtained from the hospital death certificates and previous medical history, and the coded discharge diagnosis data	age, diastolic blood pressure, systolic blood pressures, previous and current smoker, BMI, daily alcohol use, co-morbidities, season at recruitment, treatment allocation, laboratory measurements
Weinstein [34], ATBC	2018, Finland	59.5 (M)	4616 (2884)	28 y	competitive chemiluminescence immunoassay/radioimmunoassay/liquid chromatography tandem mass spectrometry	obtained from Finnish Cancer Registry	BMI, number of cigarettes smoked per day, years of smoking, physical activity, serum cholesterol, history of diabetes, family history of cancer, systolic blood pressure, trial intervention group, and calendar year of diagnosis
Cawthon [49], MrOS	2010 USA	73.7 (M)	1490 (97)	7.3 y	chemiluminescence immunoassay	obtained from six U.S. clinical centers through death certificates and discharge summaries	age, clinic, season of blood collection, serum calcium and phosphate, GFR, percentage body fat, weight, race, health status, presence of at least one medical condition, alcohol use, education, activity level, marital status, and presence of a functional or mobility limitation
Hutchinson [42], TS	2010 Norway	Nonsmoker 61 Smoker 57.2 (Both)	7161 (498)	13 y	mass spectrometry	obtained from Norway Cancer Registry	age, gender, season, BMI, physical activity score, diabetes, hypertension, serum creatinine, prior cardiovascular disease and prior cancer
Freedman [51], NHANES III	2010 USA	44 (Both)	16,819 (884)	12.5 y	electrochemiluminescence immunoassay	obtained from National Center for Health Statistics of the Centers for Disease Control and Prevention	age, race/ ethnicity, smoking history, and BMI.
Pilz [52], LURIC	2008 Germany	62.7 (Both)	3257 (95)	7.75 y	radioimmunoassay	obtained from local person registries	age, gender, season, BMI, active smokers, retinol, exercise tertiles, beer and wine consumption, and diabetes mellitus.
Eaton [53], WHI	2011 USA	65.8 (F)	2429 (62)	8 y	high-performance liquid chromatography	obtained from the Women’s Health Initiative	age, season, ethnicity, CaD trial indicator, education, smoking status, current aspirin use, history of fracture, waist circumference, BMI, physical activity, and use of vitamin D supplements.
Skaaby [54], Monica10 and Inter99.	2012 Denmark	49.8 (Both)	9146 (301)	10 y	chemiluminescence immunoassay	obtained from Danish Registry of Causes of Death	study group (no intervention (participants from Monica10), lifestyle counseling (group B from Inter99), lifestyle and group counseling (group A from Inter99)), gender, education, season of blood sample, intake of fish, physical activity, smoking, BMI and alcohol consumption.
Schöttker [55], ESTHER	2013 Germany	62 (Both)	9578 (433)	9.5 y	competitive protein-binding assay	identified by inquiry at the residents’ registration offices	age, sex, season of blood draw, regularly intake of multi-vitamin supplements, fish consumption, BMI, scholarly education, physical activity, smoking, systolic blood pressure, chronic kidney disease, serum CRP concentrations and total cholesterol
Signorello [56], SCCS	2012 USA	59.5 (Both)	3704 (954)	7 y	chemiluminescence immunoassay	identified by Social Security Administration’s Death Master File and the National Death Index	gender, race, age, community health center enrollment site, date of blood collection, BMI, smoking, physical activity, and household income.
Khaw [57], -	2014 UK	62 (Both)	14,641 (1086)	13 y	mass spectrometry	obtained fromNational Cancer Registry for incident Cancer	age, sex, month, BMI, physical activity, smoking, alcohol, vitamin C, diabetes, history of cardiovascular disease, history of cancer, social class, and education
Kritchevsky [58], Health ABC	2012 US	74.7 (Both)	2638 (218)	8.5 y	immunoassay	identified by medical records, death certificates, proxy information, and autopsy reports	age, gender, race, education, season, field center, smoking status, pack years, alcohol consumption, body mass index, time walking, usual 20m walking speed, estimated glomerular filtration rate, cognition, depressive symptoms, IL-6, cholesterol, and prevalent diabetes, hypertension, cardiovascular disease, cancer, or lung disease.
Rohrmann [59], Swiss MONICA	2012	47.1 (Both)	3198 (188)	18 y	protein-bound assay	obtained from Swiss National Cohort	age, sex, sunlight exposure, systolic blood pressure, smoking status, nationality
Michaelsso [44], ULSAM	2010 Sweden	71 (M)	1194 (164)	12.7 y	high-performance liquid chromatography–tandem mass spectrometry	obtained from Swedish National Cancer Registry and Cause of Death Registry	age, weight, height, calcium intake, season of blood draw, social class, smoking status, leisure physical activity, self-perceived health, diabetes mellitus, other endocrine disease, hematologic diseases, dermatoses, infectious disease, musculoskeletal disease, psychiatric disease, respiratory disease, kidney or urinary disease, gastrointestinal disease, supplemental vitamin D use, total vitamin D intake, fish intake, plasma parathyroid hormone, plasma cystatin C, plasma C-reactive protein, serum calcium, serum phosphate, plasma troponin I, plasma N-terminal pro brain natriuretic peptide, plasma cholesterol, plasma triglycerides, plasma HDL cholesterol, plasma retinol, plasma insulin, total energy intake, and alcohol intake and systolic blood pressure, diastolic blood pressure, lipid-lowering treatment, and antihypertensive treatment.

^b^ There were 16 prospective cohort studies comprising 8729 cancer mortality cases among 10,794 participants in relation to 25-hydroxyvitamin D. EMAS: the European Male Ageing Study; GPTL: the General Population Trial of Linxian; CAIFOS: Calcuim Intake Fracture Outcome Study; ATBC: The Alpha-Tocopherol, Beta-Carotene Cancer Prevention Study; MrOS: Osteoporotic Fractures in Men study; GFR: Glomerular filtration rate; TS: Tromsø Study; NHANES III: Third National Health and Nutritional Examination Survey; LURIC: The Ludwigshafen Risk and Cardiovascular Health study; WHI: the Women’s Health Initiative; ESTHER: Epidemiological investigations of the chances of preventing, recognizing early and optimally treating chronic diseases in an elderly population; SCCS: The Southern Community Cohort Study; Health ABC: the Health, Aging, and Body Composition study ; ULSAM: The Uppsala Longitudinal Study of Adult Men; CaD: calcium and vitamin D; IL-6: interleukin-6.

## Supplementary Materials

The following are available online at https://www.mdpi.com/2072-6643/11/10/2295/s1, Figure S1: Sensitivity analysis with respect to vitamin D concentration and cancer incidence; Figure S2: Sensitivity analysis with respect to vitamin D concentration and cancer mortality; Table S1 Quality assessment of studies investigating 25(OH)D and cancer risk, according to the Newcastle-Ottawa Scale; Table S2. Quality assessment of studies investigating 25(OH)D and cancer mortality, according to the Newcastle-Ottawa Scale; Table S3: Subgroup and meta-regression analyses for cancer incidence; Table S4: Subgroup and meta-regression analyses for cancer mortality.
