# Supplementary material for: 25-Hydroxyvitamin D and Total Cancer Incidence and Mortality: A Meta-Analysis of Prospective Cohort Studies
Source: Nutrients. 2019 Sep 26;11(10):2295. doi: 10.3390/nu11102295 (PMC6835972; doi:10.3390/nu11102295)
Supplement: Supplementary file 1 [file nutrients-11-02295-s001.pdf]

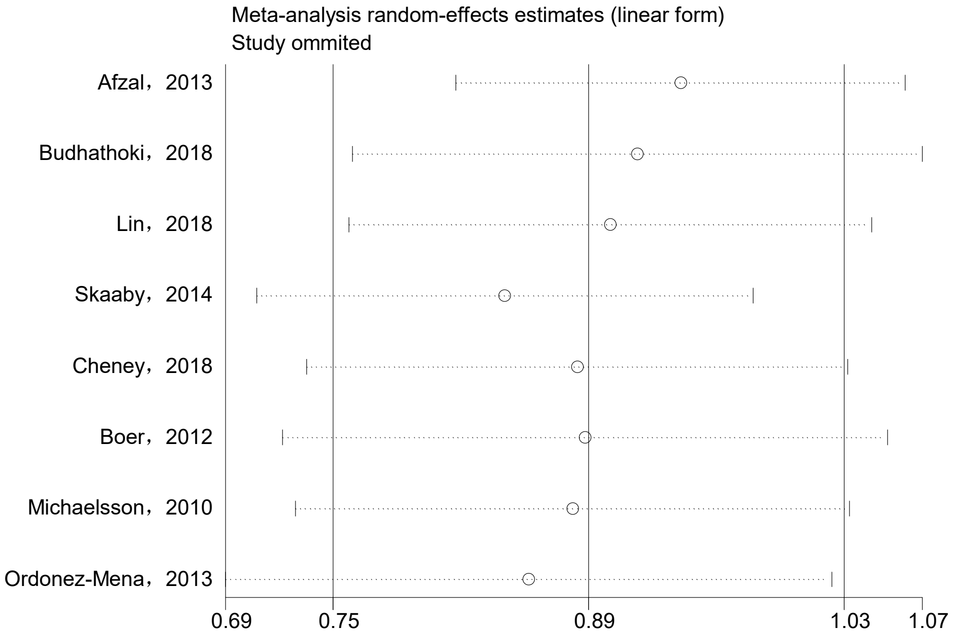

Figure S1. Sensitivity analysis with respect to vitamin D concentration and cancer incidence. The circles represent the summary effect after removing one study. On the middle of the line: the summary effect. On both sides of the line: 95%CI.

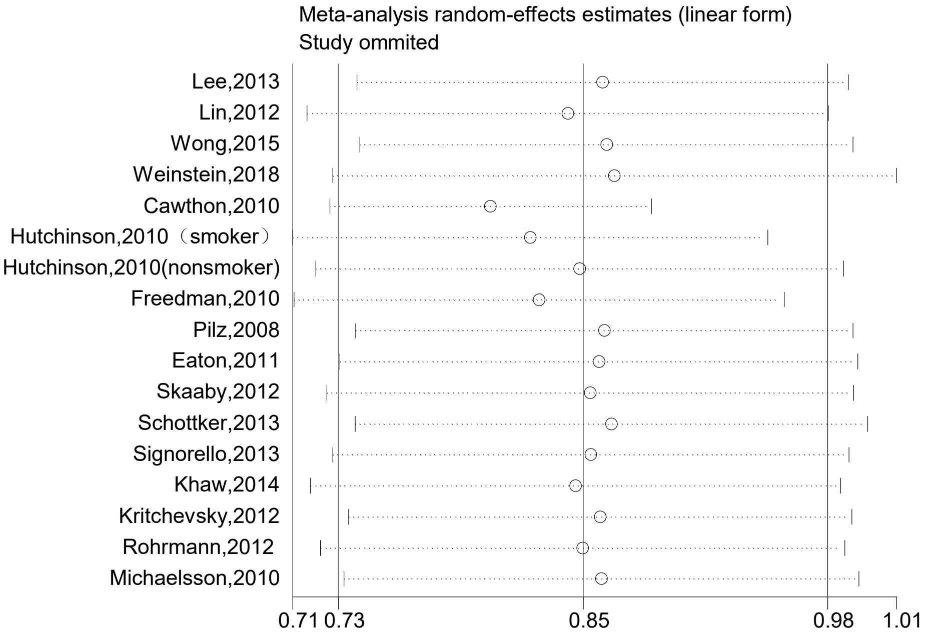

Figure S2: Sensitivity analysis with respect to vitamin D concentration and cancer mortaliy. The circles represent the summary effect after removing one study. On the middle of the line: the summary effect. On both sides of the line: 95%CI.

Table S1. Quality assessment of studies investigating 25(OH)D and cancer risk

Study	Representativeness of the exposed cohort	Selection of the unexposed cohort	Ascertainment of exposure	Demonstration that outcome of interest at start of study	Comparability of cohorts on the basis of the design or analysis	Outcome assessment	Follow-up long enough for the outcomes to occur	Adequacy of follow-up of cohorts	Total quality scores
Afzal	-	-	☆	☆	☆☆	☆	☆	☆	☆☆☆☆☆☆☆
Budhathoki	-	☆	☆	☆	☆☆	☆	☆	☆	☆☆☆☆☆☆☆
Lin	☆	☆	☆	☆	☆☆	☆	-	☆	☆☆☆☆☆☆☆☆
Skaaby	☆	-	☆	☆	☆☆	☆	☆	-	☆☆☆☆☆☆☆
Cheney	☆	-	☆	☆	☆☆	-	-	☆	☆☆☆☆☆☆
Boer	☆	-	☆	☆	☆☆	-	☆	☆	☆☆☆☆☆☆☆
Ordonez-Mena1	-	-	☆	☆	☆☆	☆	☆	☆	☆☆☆☆☆☆
Michaelsson	-	-	☆	☆	☆☆	☆	☆	-	☆☆☆☆☆☆

Table S2. Quality assessment of studies investigating 25(OH)D and cancer mortality.

Study	Representativeness of the exposed cohort	Selection of the unexposed cohort	Ascertainment of exposure	Demonstration that outcome of interest at start of study	Comparability of cohorts on the basis of the design or analysis	Outcome assessment	Follow-up long enough for the outcomes to occur	Adequacy of follow-up of cohorts	Total quality scores
Lee	-	-	☆	☆	☆☆	-	☆	☆	☆☆☆☆☆☆
Lin	☆	-	☆	☆	☆☆	☆	☆	☆	☆☆☆☆☆☆☆☆
Schottker	☆	-	☆	☆	☆☆	☆	☆	☆	☆☆☆☆☆☆☆☆
Wong	-	-	☆	☆	☆☆	☆	☆	☆	☆☆☆☆☆☆☆
Weinstein	-	-	☆	☆	☆☆	☆	☆	☆	☆☆☆☆☆☆☆
Cawthon	-	-	☆	☆	☆☆	☆	☆	-	☆☆☆☆☆☆
Hutchinson	☆	-	-	☆	☆☆	☆	☆	☆	☆☆☆☆☆☆☆

Freedman	☆	-	☆	☆	☆☆	☆	☆	-	☆☆☆☆☆☆☆
Pilz	-	-	☆	☆	☆☆	☆	☆	☆	☆☆☆☆☆☆☆
Eaton	-	-	☆	☆	☆☆	☆	☆	-	☆☆☆☆☆☆
Skaaby	☆	-	☆	☆	☆☆	☆	☆	-	☆☆☆☆☆☆☆
Krause	-	-	☆	☆	☆☆	☆	☆	-	☆☆☆☆☆☆
Signorello	☆	-	☆	☆	☆☆	☆	☆	☆	☆☆☆☆☆☆☆☆
Khaw	☆	-	☆	☆	☆☆	☆	☆	☆	☆☆☆☆☆☆☆☆
Kritchevsky	-	-	☆	☆	☆☆	☆	☆	☆	☆☆☆☆☆☆☆
Rohrmann	☆	-	☆	☆	☆☆	-	☆	☆	☆☆☆☆☆☆☆
Michaelsson	-	-	☆	☆	☆☆	☆	☆	-	☆☆☆☆☆☆

Table S3. Subgroup and meta-regression analyses for cancer incidence.

Factors stratified	NO.	Pooled relative risk (95% CI)	Heterogeneity		Pb
			I^2 (%)	Pa	0.275
region					0.664
Europe	5	0.91(0.71, 1.16)	76.0%	0.002	
Asia	2	0.77(0.66, 0.89)	0.0%	0.341	
US	1	0.88(0.76, 1.11)	-	-	
Study quality					0.284
High quality	5	0.80(0.63, 1.01)	80.2%	0.001	
medium quality	3	1.01(0.88, 1.18)	0.0%	0.989	
Gender					0.168
Male	3	0.69(0.47, 1.00)	73%	0.025	
Female	2	0.80(0.35, 1.82)	75.8%	0.042	

Table S4. Subgroup and meta-regression analyses for cancer mortality.

Factors stratified	NO.	Pooled relative risk (95% CI)	Heterogeneity		Pb
			I2 (%)	Pa	
region					0.616
Europe	8	0.79(0.65, 0.90)	38.1%	0.104	
Asia	1	0.96(0.66,1.39)	-	-	
US	7	0.83(0.56, 1.21)	66.6%	0.011	
gender					0.942
male	6	0.90(0.73, 1.12)	82.8%	0.001	
female	5	0.72(0.52, 0.98)	56.6%	0.075	
Study quality					0.852
High quality	11	0.82(0.71, 0.95)	42.7%	0.058	
medium quality	5	0.78(0.52, 1.18)	66.8%	0.988	

CI., confidential interval; NO., number of included studies. Pa for heterogeneity Pb for meta-regression analysis
